# Hypovitaminosis D in patients with oral leukoplakia: insights from a cross-sectional study

**DOI:** 10.3389/fonc.2025.1522726

**Published:** 2025-02-06

**Authors:** Andrea Maturana-Ramirez, Juan Aitken-Saavedra, Dante Mora-Ferraro, Gabriel Rojas-Zúñiga, Iris Espinoza-Santander, Gonzalo Rojas-Alcayaga, Ana Ortega-Pinto, Montserrat Reyes, Diego Lazo, Egardo Caamanão

**Affiliations:** ^1^ Department of Oral Pathology and Medicine, Faculty of Dentistry, Universidad de Chile, Santiago, Chile; ^2^ Therapeutic Diagnostic Center Odontology and Pathological Anatomy Service, Hospital Complex San Jose, Santiago, Chile; ^3^ Dentist, Faculty of Dentistry, Universidad de Chile, Santiago, Chile; ^4^ Laboratory of Endocrinology and Reproductive Biology of the Clinical Hospital, University of Chile, Santiago, Chile

**Keywords:** vitamin D, calcitriol, oral leukoplakia, oral cancer, oral squamous cell carcinoma, smoker

## Abstract

**Introduction:**

Oral leukoplakia is one of the most frequent oral potentially malignant disorders. The present study aims to compare serum vitamin D levels between patients with and without oral leukoplakia, by smoking habit.

**Methods:**

This cross-sectional study involved a group of 45 cases with oral leukoplakia and a control group with 45 individuals. In both groups a pathology report was done, and for leukoplakia a binary classification of low- and high-grade epithelial dysplasia was employed. Serum 25(OH)D3 vitamin D levels, and data on smoking status, age, gender, comorbidities, and clinical and pathological characteristics were collected for both groups.

**Results:**

vitamin D levels were lower in the oral leukoplakia group with a median of 19.1 ng/ml, while the control group had a median of 24.8 ng/ml. When subdividing each group by smoking habit, the smoking case group had a median of 19.4 ng/ml (IQR: 15.7-21.5 ng/ml), the non-smoking case group had 18.8 ng/ml (IQR: 13.6-29.2 ng/ml), the smoking control group had 21.8 ng/ml (IQR: 17.5-27.3 ng/ml), and the non-smoking control group had 25.4 ng/ml (IQR: 20.4-32.9 ng/ml) (p<0.05). When comparing serum vitamin D levels, statistically significant differences were found between the smoking case group versus the non-smoking control group and between the non-smoking case group versus the non-smoking control group (p<0.05). Serum vitamin D levels by histopathological diagnosis showed no differences between leukoplakia groups.

**Discussion:**

This study shows that serum vitamin D levels were lower in patients with OL compared to those without OL, which was more evident in the smoking group. Patients with OL were previously observed to have hypovitaminosis D, without assessing smoking habits. This finding suggests a possible role of vitamin D deficiency in the development of OL, which could be more marked in smokers. This opens the possibility of future research on vitamin D as a chemopreventive agent in the malignant transformation of OL, and to evaluate the relationship between smoking and hypovitaminosis D.

## 1 Introduction

Oral squamous cell carcinoma (OSCC) represents the most common malignant neoplasm in the oral cavity, accounting for approximately 90% of all oral cancer cases (1). It is characterized by high aggressiveness and a low five-year survival rate, as well as a significant impact on patients’ quality of life (2, 3). The development of OSCC is understood as a multistep process influenced by endogenous and exogenous factors, triggering complex molecular changes (4). A high percentage of OSCC cases are estimated to develop from precursor lesions, known as oral potentially malignant disorders (OPMD) (5).

Oral leukoplakia (OL) is one of the most frequent OPMD and is defined as a predominantly white plaque on the oral mucosa that cannot be characterized as any other known condition, with an increased risk of malignancy. It is classified into homogeneous and non-homogeneous clinical subtypes, with the latter carrying a higher risk of malignant transformation (6). The estimated rate of malignant transformation for OL varies between 1.1% and 40.8%, with a weighted proportion of 9.8% (7). Among OPMDs, proliferative verrucous leukoplakia (PVL) is a clinicopathological subtype of OL, defined as: “A progressive, persistent, and irreversible disorder characterized by the presence of multiple leucoplakias that frequently become verrucous” (6). It has a high recurrence rate and a significant risk of progression to OSCC (8). The weighted proportion of malignant transformation is estimated at 43.87%, making it the highest-risk disorder among OPMDs (9).

Vitamin D is a steroid hormone with multiple systemic functions and extra-skeletal effects. It has been extensively studied in the context of various neoplasms, with numerous anticancer properties described, including antiproliferative effects, induction of apoptosis, and modulation of inflammation (10–12). In head and neck cancers, a high prevalence of vitamin D deficiency has been observed, and an inverse correlation between serum levels of this vitamin and the risk of developing oral cancer has been documented (13, 14). Additionally, *in vitro* studies have demonstrated that calcitriol, the active form of vitamin D, can inhibit the growth of OSCC cell lines (15). However, the relationship between vitamin D levels and OPMDs, particularly OL, has yet to be fully elucidated.

Given the complexity and heterogeneity of OPMDs, and the absence of fully effective therapeutic alternatives to prevent their malignant transformation (16), it is relevant to consider vitamin D as a potential chemopreventive agent in their management. Recent studies suggest that adequate vitamin D levels may regulate the immune response and reduce the risk of progression to OSCC (17, 18). A recent clinical study compared serum vitamin D levels in patients with OL and healthy controls, finding a significant difference that supports the hypothesis of a relationship between vitamin D deficiency and OL development (19). However, to date, this relationship has not been investigated in the Chilean population, where risk factors such as betel use, predominant in Asia, are absent (20). The present study aims to compare serum vitamin D levels between patients with and without oral leukoplakia, providing a foundation for future research on the role of vitamin D in the prevention and management of this condition.

## 2 Materials and methods

### 2.1 Design and sample

This cross-sectional study included two groups of adult participants: a case group with OL and a control group without OL or OSCC, though individuals in the control group may have had other diagnoses like mucoceles, fibromas, or other lesions. The sample size, consisting of 45 cases and 45 controls, was determined with a significance level of 5% and a desired statistical power of 0.9, making it adequate and significant for the objectives of the study.

The inclusion criteria for both groups were an age of 18 years or older and a pathology report performed by an oral and maxillofacial pathology specialist. For the OL group, additional inclusion criteria included a clinical diagnosis of leukoplakia with a pathology report aligned with the 2017 WHO criteria (1). These pathology reports employed a binary classification of low- and high-grade epithelial dysplasia (21), requiring consensus between two oral and maxillofacial pathology specialists (AM and AO). In cases of discrepancy, a third pathologist (IE or GR) contributed to resolve the degree of dysplasia.

Exclusion criteria in both groups were previous diagnosis of OSCC or OPMDs, patients irradiated in the head and neck area, those with terminal illnesses, severe neurological damage, mental disorders, liver or kidney failure, pregnancy, immunological diseases, or those who had taken vitamin D supplements in the past 6 months.

The patients were sourced from the Oral Medicine Clinic of the San Jose Hospital and Faculty of Dentistry at the University of Chile, in the north Metropolitan area in Santiago, Chile.

Serum vitamin D 25(OH)D3 levels were obtained in all the participants. Information about smoking habits (smokers and non-smokers), IPA age, gender, comorbidities and clinical and pathological characteristics in case with OL were obtained at the time of the inclusion in the study.

Ethical approval for the research project was obtained from the Ethics Committee of the North Metropolitan Health Service in Santiago of Chile (063/2019, august 2019), and the study adhered to the recommendations of the Declaration of Helsinki (21). All participants provided written informed consent before their involvement in the study.

### 2.2 Determination of serum vitamin D level 25(OH)D3 levels

All patients underwent serum calcidiol (25(OH)D3) level measurements, conducted at the Clinical Laboratory of the Clinical Hospital of the University of Chile. A fasting blood sample (minimum 1 mL, with a minimum serum volume of 500 µL) was obtained and analyzed in the Endocrinology Laboratory of the same hospital. The electrochemiluminescence method, employing a standardized technique on the Cobas 601 equipment (ROCHE HITACHI), was used for analysis. The classification of 25(OH)D3 levels was as follows: Sufficiency (>35 ng/ml), Mild deficiency (25-35 ng/ml), Moderate deficiency (12.5-24.9 ng/ml), and Severe deficiency (<12.5 ng/ml) (4).

### 2.3 Determination of smoking habit

The cigarette-smoking status of participants was assessed through a self-administered questionnaire. None of the study participants reported using electronic cigarettes or smokeless tobacco products. Smoking status was categorized in the questionnaire as follows: ‘smoker,’ defined as an individual who had smoked at least 100 cigarettes in total since initiation of smoking, and ‘nonsmoker,’ defined as an individual who had either never smoked or had smoked fewer than 100 cigarettes in total since initiation of smoking (22). With these data, the pack-year index (PYI) was calculated, defined as the daily number of cigarettes multiplied by the years of consumption, and divided by 20, which is the number of cigarettes in a pack, to be used as an indicator of a person’s total consumption (23, 24).

### 2.4 Statistical analysis

Descriptive statistics were reported as medians and interquartile ranges, as the Shapiro-Wilk test indicated a non-normal distribution of the data. Comparisons between two groups were performed using the Mann-Whitney U test, while comparisons among subgroups (e.g., smoking and non-smoking control groups, smoking and non-smoking case groups, and subgroups within the case group based on histopathological diagnosis) were conducted using the Kruskal-Wallis test. To identify specific group differences, Dunn’s multiple comparison method was applied. For categorical variables within the same population (e.g., gender, smoking status, and comorbidities), between case patients and controls, a Chi-squared test was used. Statistical significance was defined as p ≤ 0.05. Analyses were performed using R software version 4.3.1 and STATA 16.0.

#### 2.4.1 Methodological approach for multivariate regression analysis

The multivariate analyses, with GLM command in Stata was used with a modified Poisson approach to estimate the prevalence ratio and confidence intervals using robust error variances. Leucoplakia (yes/no) was modeled with serum vitamin D, age, sex, and smoking habit(yes/no).

## 3 Results

### 3.1 Clinical and epidemiological characterization of the total sample

This study was based on a total sample of 90 individuals, comprising a group of 45 cases with OL and another group of 45 controls without OL, treated at the San José Hospital and the Oral Medicine Clinic of the Faculty of Dentistry at the University of Chile, between 2018 and 2023. The total sample (n = 90) consisted of 57 women (63.3%) and 33 men (36.6%). The case group (n = 45) was made up of 27 women (60%) and 18 men (40%), while the control group included 30 women (66.6%) and 15 men (33.3%). The sex distribution did not show statistically significant differences between cases and controls (p>0.05). The participants’ ages ranged from 21 to 83 years, with a median age of 61 years (IQR: 54-69 years) in the total sample; 59 years (IQR: 53-68 years) in the case group, and 62 years (IQR: 54-69 years) in the control group. There was no statistically significant difference in age when comparing cases and controls (p>0.05). Additionally, 84.4% of the cases and 91.1% of the controls were 50 years or older.

The most prevalent diseases in the total sample were hypertension (HTN) and diabetes mellitus (DM). HTN had a prevalence of 51.2% in the cases and 51.4% in the controls, with no statistically significant differences (p>0.05). DM had a prevalence of 22% in the cases and 20% in the controls, also without statistically significant differences (p>0.05). Other pathologies present in the total sample included dyslipidemia (14.8%) and hypothyroidism (11.8%).

Regarding tobacco use, from the total sample (n=90), data on this habit were unavailable for 7 individuals, corresponding to 2 cases and 5 controls, so this variable was described in a sample of 83 individuals, divided into 43 cases and 40 controls. From this sample (n=83), it was identified that 50.6% were smokers. When analyzed by study groups, 65.1% of the cases were smokers, while 35% of the controls were smokers, with statistically significant differences (p<0.05). Regarding the PYI, the median for smokers in the case group was 14.5 PYI (IQR: 7.1-29.9 PYI), while in the control group, it was 4.5 PYI (IQR: 1.9-25.8 PYI), with statistically significant differences (p<0.05).

### 3.2 Clinical and pathological characterization of patients with oral leukoplakia

The most common location of OL was gingiva (51.1%), followed by tongue (24.4%) and buccal mucosa (17.8%). Regarding clinical diagnosis, non-homogeneous OL was the most frequent (46.7%), followed by PVL (31.1%). In terms of histopathological diagnosis, low-grade dysplasia (LGD) was the most prevalent (55.5%) (**Tables 1**, **2**).

**Table 1:** Clinicopathological characteristics of patients with oral leukoplakia.

Clinicopathological characteristics	n (%)
Lesion location	
Gingiva Tongue Buccal Mucosa Hard palate Retromolar trigone Floor of mouth Total	23 (51,1) 11 (24,4) 8 (17,8) 2 (4,5) 1 (2,2) 0 (0) 45 (100)
Clinical diagnosis	
Nonhomogeneous leukoplakia Proliferative verrucous leukoplakia Homogeneous leukoplakia Total	21 (46,7) 14 (31,1) 10 (22,2) 45 (100)
Histopathological diagnosis	
Low grade epithelial dysplasia Hyperplasia with hyperkeratosis High grade epithelial dysplasia Total	25 (55,5) 12 (26,7) 8 (17,8) 45 (100)

**Table 2:** Clinical and epidemiological characterization of the total sample.

Variable	OL n (%)	Controls n (%)	p value
Sex			
Women	27 (60)	30 (66.7)	0.662
Men	18 (40)	15 (33.3)	
Age			
<50 years	7 (15.6)	4 (8.9)	0.519
≥50 years	38 (84.4)	41 (91.1)	
Smoking habit^a^			
Yes	28 (65.1)	26 (35)	0.012
No	15 (34.9)	14 (65)	
Pack-Year Index			
Median	14.5	4.5	0.001
Interquartile range	7.1-29.9	1.9-25.8	
Comorbidities^b^			
Hypertension	21 (51.2)	18 (51.4)	1
Diabetes Mellitus	9 (22)	7 (20)	1
Dyslipidemia	4 (9.8)	7 (20)	0.348
Hypothyroidism	5 (12.2)	4 (11.4)	1
Total	45 (100)	45 (100)	

a 2 missing data in OL and 5 missing data in Controls.

b 4 missing data in OL and 10 missing data in Controls.

OL, Oral Leukoplakia

### 3.3 Comparison of serum vitamin D levels between cases and controls

In the case group, only 4.4% (n = 2) of individuals had normal vitamin D levels, compared to 13.3% (n = 6) in the control group. Mild deficiency was observed in 13.4% (n = 6) of the cases and 35.6% (n = 16) of the controls. Moderate deficiency was predominant in both groups, affecting 71.1% (n = 32) of the cases and 51.1% (n = 23) of the controls. Finally, severe deficiency was reported in 11.1% (n = 5) of the cases, while no cases of severe deficiency were recorded in the control group.

**Figure 1:**
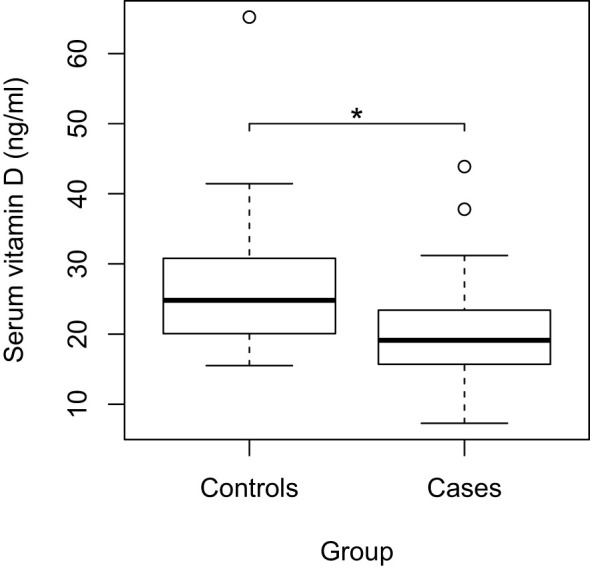
Comparison of serum vitamin D levels between Cases and Controls; *p<0,05; circles: outliers.

The total sample had a median serum vitamin D level of 21.4 ng/ml (IQR: 17.7-27.2 ng/ml). The case group had a median of 19.1 ng/ml (IQR: 15.4-23.8 ng/ml), while the control group had a median of 24.8 ng/ml (IQR: 20-30.9 ng/ml) (**Figure 1**). When comparing vitamin D levels between cases and controls, a statistically significant difference was found (p<0.05).

### 3.4 Comparison of serum vitamin D levels between cases and controls by smoking habit

**Figure 2:**
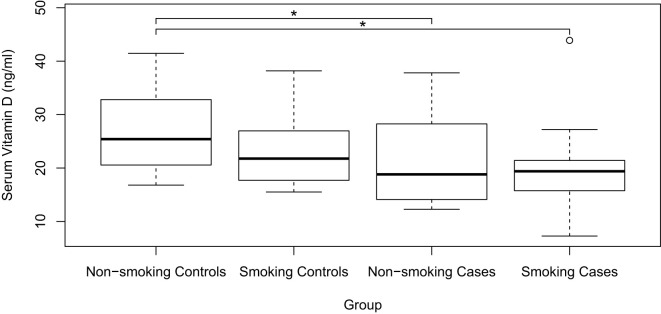
Comparison of serum vitamin D levels between Smoking and Non-smoking Cases and Controls; *p<0,05; circles: outliers. Additionally, when no brackets are present in the graph, it indicates that there are no statistically significant differences between the groups.

When subdividing each group by smoking habit, the smoking case group had a median of 19.4 ng/ml (IQR: 15.7-21.5 ng/ml), the non-smoking case group had 18.8 ng/ml (IQR: 13.6-29.2 ng/ml), the smoking control group had 21.8 ng/ml (IQR: 17.5-27.3 ng/ml), and the non-smoking control group had 25.4 ng/ml (IQR: 20.4-32.9 ng/ml) (**Figure 2**). When comparing serum vitamin D levels, statistically significant differences were found between the smoking case group versus the non-smoking control group and between the non-smoking case group versus the non-smoking control group (p<0.05).

### 3.5 Comparison of serum vitamin D levels among histopathological diagnoses in patients with oral leukoplakia

In the case group (n=45), serum vitamin D levels were analyzed according to different histopathological diagnoses. In the hyperplasia with hyperkeratosis (HK) group, none of the cases had normal vitamin D levels, while in the low-grade dysplasia (LGD) and high-grade dysplasia (HGD) groups, normal levels were observed in 1 case each. Mild deficiency affected 2 cases with HK, 3 with LGD, and 1 with HGD. Moderate deficiency was the most prevalent, detected in 8 cases with HK, 20 with LGD, and 4 with HGD. Finally, severe deficiency was observed in 2 cases with HK, 1 with LGD, and 2 with HGD.

Regarding serum vitamin D levels, the HK group had a median of 19.7 ng/ml (IQR: 15.3-23.6 ng/ml), the LGD group had 19.7 ng/ml (IQR: 15.2-23.8 ng/ml), and the HGD group had 18.6 ng/ml (IQR: 13.2-27.8 ng/ml). When comparing serum vitamin D levels by histopathological diagnosis, no statistically significant differences were found between the case groups (p>0.05).

Multivariate regression analysis showed that smoking was not a significant confounder in the association between serum vitamin D levels and OL. The association between lower vitamin D levels and OL appears to be independent of smoking status (**Table 3**).

**Table 3:** Multivariate regression analysis.

Variable	RR (95% CI)	p value
Sex (female)^a^	1.05 (0.56 – 1.95)	0.877
Age (<50 years)^b^	0.98 (0.95 – 1.01)	0.209
Serum vitamin D level	0.95 (0.91 – 0.99)	0.047
Smoking habit (yes)^c^	1.57 (0.81 – 3.03)	0.178

a Reference category = male.

b Reference category = ≥50 years.

c Reference category = no

## 4 Discussion

The objective of this study was to compare serum vitamin D levels between patients with and without OL. In terms of sex distribution in the total sample, there was a similar distribution between cases and controls, with most female participants. This result aligns with a previous study conducted at the Faculty of Dentistry, University of Chile, which evaluated patients with various OPMD and oral cancer, finding that 60% were women (25). The higher proportion of women in the total sample contrasts with literature indicating a tendency for higher OL development in men, although an increased risk of malignant transformation in women has been reported (7, 26). This higher number of women in our sample could be due to women consulting health services more frequently than men (27), facilitating diagnosis of these lesions; however, further studies are needed to confirm this in the Chilean context. The age profile of the total sample aligns with that described in the literature, as most participants in this study were 50 years or older, with similar age values observed when comparing cases and controls. This finding is like a study that reported 70.9% of OL subjects aged 50 years or older in its sample (28).

The total sample studied showed a high prevalence of non-communicable chronic diseases such as HTN and DM, like the most recent statistics from the Chilean National Health Survey (NHS) conducted between 2016-2017. In this sample, 51.3% had HTN, comparable to the NHS 2016-2017 findings, which reported that 45.1% of the population aged 45-64 years has this condition (29). On the other hand, 21.1% of the total sample had DM, aligning with the NHS 2016-2017 result indicating that 18.3% of the population aged 45-64 years has this disease (30). The prevalences of HTN and DM were similar between case and control groups, suggesting that differences in serum vitamin D levels between the two groups are unlikely to be influenced by this variable. Evidence suggests an inverse relationship between vitamin D levels and the risk of pre-HTN and HTN, explained by mechanisms linking vitamin D deficiency to renin-angiotensin system activation, vascular endothelial dysfunction, disruptions in calcium homeostasis, and free radical production (31). Additionally, long-term studies have shown an inverse correlation between vitamin D levels and the occurrence of DM, a relationship mediated mainly by the anti-inflammatory effect of vitamin D, as well as pathways through which it directly enhances insulin sensitivity and secretion (32).

Regarding tobacco consumption, a high prevalence of smokers was identified in the total sample. The proportion of smokers among controls is consistent with the latest NHS 2016-2017, which reported that 32.5% of the Chilean population are smokers (33); however, the percentage of smokers in the case group was significantly higher than that in the control group. This trend of higher tobacco use among cases is noteworthy and aligns with the fact that tobacco is considered one of the main risk factors for the development of OL and OSCC (8, 34). Furthermore, evidence suggests that smokers have lower serum vitamin D levels, regardless of supplementation (35). In line with this, in a 28-year prospective study, lower plasma levels of 25(OH)D were found to be associated with an increased risk of tobacco-related cancers, but not with the risk of other cancers (36). This underscores the importance of addressing smoking cessation as part of the comprehensive management of patients with OL and assessing vitamin D levels among smokers.

The most prevalent locations of OL in this study were gingiva and tongue, consistent with the results of a population cohort study conducted in the United States that evaluated 500 patients with OL (28). This finding is relevant considering that both sites are among the most affected by OC, and the tongue is described as one of the locations with the highest risk of malignant transformation for OL (8).

Regarding the clinical diagnoses of OL, a higher prevalence of non-homogeneous OL versus homogeneous OL was found, differing from a meta-analysis reporting that the global prevalence of homogeneous OL is greater than that of non-homogeneous OL (26). This finding is significant, as the patients in our sample may have a higher risk of malignant transformation. On the other hand, this may be attributed to the convenience sampling method used in this study, in which most of the patients treated may have been prioritized for care due to having a non-homogeneous subtype of OL with more notable clinical manifestations.

The most prevalent histopathological diagnoses were LGD and HK, like the previously mentioned U.S. population cohort study (28). It is important to note that while the degree of ED is one of the main risk factors for malignant transformation in OL, HK or LGD in this case does not imply an absence of malignant transformation risk; therefore, patients should still undergo regular evaluations (6).

The high prevalence of vitamin D deficiency (<35 ng/ml) found in the total sample, is consistent with previous studies in Chile (37, 38). We believe that these findings highlight the need to address vitamin D deficiency in Chile, especially among older adults, a demographic data that also shows a higher prevalence of OSCC (39). A study based on the NHS 2016-2017 determined that, among Chile’s older adult population, determinants associated with a greater likelihood of vitamin D deficiency are the following conditions: women, people of native origin, urban dwellers, shorter sunlight exposure, and greater geographical latitude. Consequently, promotion and prevention programs should primarily target older adults living in urban areas in southern Chile, with a particular focus on women (40). In our results, the total sample presented moderate deficiency, and although most participants had vitamin D deficiency, statistically significant differences were found when comparing cases and controls. This finding supports the hypothesis of this study and is consistent with previous research (19).

Regarding the comparison of serum vitamin D levels among histopathological diagnosis, moderate deficiency predominated, with no significant differences observed. The literature does not provide similar studies for comparative analysis in this regard. It is possible that vitamin D deficiency favors the development of OL.

A study evaluated an *in vivo* mouse model with chronic vitamin D deficiency, which showed increased oral epithelial proliferation but no development of morphological or histological abnormalities in the oral epithelium. The authors suggested that vitamin D deficiency alone is insufficient to disrupt oral epithelial homeostasis and induce carcinogenesis (41). However, recent findings highlight the therapeutic potential of calcitriol in preventing the progression of lesions with ED to OSCC (42), and other studies indicate a possible role of vitamin D deficiency in the progression of OPMD to OSCC (43, 44).

The difference found in serum vitamin D levels between patients with and without OL may be comparable to previous studies demonstrating that vitamin D deficiency is linked to an increased risk of the onset and progression of lesions with malignant potential in the colon (45), as well as various cancers (46, 47) and OSCC (12, 48). Based on the presented evidence, vitamin D may play an important chemopreventive role, likely determined by its functions such as apoptosis induction and antiproliferative effects (11, 12), immunomodulatory properties, relationship with p53 signaling (49), oxidative stress inhibition (50). Therefore, vitamin D administration should be considered as a possible adjuvant tool in preventing the development of OL and its malignant transformation. Considering these results, it is suggested that serum vitamin D levels may play a still not fully elucidated role in OL development.

In managing vitamin D deficiency, the Endocrine Society, a global endocrinology community, emphasizes the importance of assessing vitamin D levels and supplementation. Vitamin D deficiency should be considered multifactorial in etiology, potentially explained by factors such as reduced synthesis or intake, decreased intestinal absorption, or conditions that impair clinical metabolism and function. Generally, there is no consensus on vitamin D supplementation protocols, which should be personalized based on treatment goals and factors such as age group, presence of systemic diseases, and body mass (51). The Spanish Society of Geriatrics and Gerontology (SEGG) recommends, for older adults with vitamin D deficiency (10-30 ng/ml), administering vitamin D3 in either spaced doses of 25,000 to 50,000 IU monthly or continuous doses of 1,000 to 2,000 IU daily, monitoring serum vitamin D levels 3-4 months after treatment initiation, and once desired levels are reached, monitoring every 6-12 months, following these general recommendations: Ensure 15-20 minutes of daily sun exposure with SPF 15-30 sunscreen; consume foods providing at least 800 IU of vitamin D daily; engage in moderate physical exercise (52).

Regarding the management of patients with OL, it is essential to conduct an accurate diagnosis, always evaluating the entire oral mucosa. It is important to consider and advise on risk factors, both lifestyle-related and those identified through clinical-pathological findings, selecting the appropriate intervention for each patient. Lifelong follow-up and periodic monitoring are necessary, along with counseling and reinforcement to support the cessation of smoking and alcohol consumption. Additionally, it is recommended to encourage the daily intake of five servings of fresh fruits and vegetables rich in antioxidants, which may help reduce risks (53), and to educate patients on self-examination, as well as when and where to seek professional evaluation.

Considering the evidence supporting the chemopreventive role of vitamin D and the results of this study, which show lower serum vitamin D levels in patients with OL compared to control patients without OL, we consider it relevant in clinical dental practice to assess, treat, and manage patients with vitamin D deficiency, with a particular focus on those with OL. Additionally, it should be noted that tobacco use, a risk factor for OSCC and OL, is associated with low vitamin D levels, which may compromise its chemopreventive role and promote the development of OC. Given the high tobacco consumption observed in the cases in this study, the importance of providing counseling and smoking cessation strategies for managing these patients is emphasized, along with routine assessment of their serum vitamin D levels.

The cross-sectional design of this study establishes an association between serum vitamin D levels and the presence or development of OL. However, this design does not allow for establishing causality, as the lack of a temporal framework prevents determining whether exposure to one variable precedes the other. Clarifying the potential chemopreventive role of vitamin D in OL development is crucial, especially considering its malignant potential and the absence of a fully effective current treatment. Therefore, further research is needed to confirm this relationship, along with studies using immunohistochemistry to evaluate markers such as p53, β-catenin, and VDR, among others. Longitudinal, clinical, case-control studies incorporating immunohistochemistry techniques are suggested, paving the way to assess vitamin D supplementation protocols for patients with OL and OPMD.

The results of the multivariate analysis indicate an association between serum vitamin D levels and the occurrence of OL. In this study, smoking was not found to be significantly associated with the occurrence of OL, highlighting the importance of evaluating serum vitamin D levels as part of the clinical assessment for patients with OL.

These results, from a global point of view, highlight the need for broader public health interventions addressing vitamin D deficiency, which may have implications for the prevention of conditions with malignant potential worldwide. For international readers, this study emphasizes the importance of understanding environmental and nutritional factors, such as vitamin D levels and tobacco use, in the context of malignant potential. The strengths of this study lie in its robust comparison of vitamin D levels among OL patients with different histopathological diagnoses, however, its limitations include the cross-sectional nature of the analysis, which precludes establishing causality, and the lack of data on other potential confounders, such as sun exposure and dietary habits.

Future studies should consider longitudinal designs to investigate the causal relationship between vitamin D deficiency and OL progression and evaluate the potential of vitamin D supplementation as a chemopreventive strategy. Specifically, prospective studies could be designed to assess the impact of vitamin D supplementation in patients with OL, evaluating outcomes such as regression, progression, or stable disease during follow-up and correlating these with serum vitamin D levels. Furthermore, exploring gene-environment interactions, in relation to tobacco use, may provide deeper insights into the mechanisms underlying the development of OL and its potential for malignant transformation.

In conclusion, this study demonstrates that serum vitamin D levels were lower in patients with OL compared to those without OL, with no differences related to histopathological diagnosis. This finding aligns with previous findings and suggests a potential role of vitamin D deficiency in the development of OL, with tobacco possibly acting as a synergistic agent. These results highlight the importance of considering vitamin D deficiency as a modifiable risk factor and open the possibility of further research into vitamin D as a chemopreventive agent in the malignant transformation of OL.

## Data Availability

The raw data supporting the conclusions of this article will be made available by the authors, without undue reservation.
